# A Genomic Snapshot of the SARS-CoV-2 Pandemic in the Balearic Islands

**DOI:** 10.3389/fmicb.2021.803827

**Published:** 2022-01-12

**Authors:** Carla López-Causapé, Pablo A. Fraile-Ribot, Santiago Jiménez-Serrano, Gabriel Cabot, Ester del Barrio-Tofiño, M. Carmen Prado, Juana María Linares, Aranzazu López, Adoración Hurtado, Elena Riera, Antoni Serra, Eva Roselló, Lluis Carbó, M. Victoria Fernández-Baca, Carmen Gallegos, Juan Saurina, Emilio Arteaga, M. Magdalena Salom, Antonia Salvá, Antoni Nicolau, Fernando González-Candelas, Iñaki Comas, Antonio Oliver

**Affiliations:** ^1^Servicio de Microbiología y Unidad de Investigación, Hospital Universitario Son Espases, Instituto de Investigación Sanitaria de las Islas Baleares, Palma, Spain; ^2^CIBER en Enfermedades Infecciosas (CIBERINFEC), Madrid, Spain; ^3^Instituto de Biomedicina de Valencia, Valencia, Spain; ^4^Servicio de Microbiología, Hospital Can Misses, Ibiza, Spain; ^5^Servicio de Microbiología, Hospital de Manacor, Manacor, Spain; ^6^Servicio de Microbiología, Hospital Mateu Orfila, Mahón, Spain; ^7^Servicio de Microbiología, Hospital Universitari Son Llàtzer, Palma, Spain; ^8^Servicio de Microbiología, Hospital Comarcal de Inca, Inca, Spain; ^9^Servicio de Epidemiología de las Islas Baleares, Palma, Spain; ^10^Gabinete Técnico-Asistencial, Servicio de Salud de las Islas Baleares, Palma, Spain; ^11^Unidad Mixta de Investigación “Infección y Salud Pública” FISABIO-Universidad de Valencia, Instituto de Biología Integrativa de Sistemas (I2SysBIO, CSIC-UV), Valencia, Spain; ^12^CIBER en Epidemiología y Salud Publica (CIBERESP), Madrid, Spain

**Keywords:** SARS-CoV-2, genomic evolution, variants, genomic epidemiology, spike mutations

## Abstract

**Objective:** To analyze the SARS-CoV-2 genomic epidemiology in the Balearic Islands, a unique setting in which the course of the pandemic has been influenced by a complex interplay between insularity, severe social restrictions and tourism travels.

**Methods:** Since the onset of the pandemic, more than 2,700 SARS-CoV-2 positive respiratory samples have been randomly selected and sequenced in the Balearic Islands. Genetic diversity of circulating variants was assessed by lineage assignment of consensus whole genome sequences with PANGOLIN and investigation of additional spike mutations.

**Results:** Consensus sequences were assigned to 46 different PANGO lineages and 75% of genomes were classified within a VOC, VUI, or VUM variant according to the WHO definitions. Highest genetic diversity was documented in the island of Majorca (42 different lineages detected). Globally, lineages B.1.1.7 and B.1.617.2/AY.X were identified as the 2 major lineages circulating in the Balearic Islands during the pandemic, distantly followed by lineages B.1.177/B.1.177.X. However, in Ibiza/Formentera lineage distribution was slightly different and lineage B.1.221 was the third most prevalent. Temporal distribution analysis showed that B.1 and B.1.5 lineages dominated the first epidemic wave, lineage B.1.177 dominated the second and third, and lineage B.1.617.2 the fourth. Of note, lineage B.1.1.7 became the most prevalent circulating lineage during first half of 2021; however, it was not associated with an increased in COVID-19 cases likely due to severe social restrictions and limited travels. Additional spike mutations were rarely documented with the exception of mutation S:Q613H which has been detected in several genomes (*n* = 25) since July 2021.

**Conclusion:** Virus evolution, mainly driven by the acquisition and selection of spike substitutions conferring biological advantages, social restrictions, and size population are apparently key factors for explaining the epidemic patterns registered in the Balearic Islands.

## Introduction

In late 2019, several cases of pneumonia of unknown etiology were detected in Wuhan in the Hubei province of China and reported to the World Health Organization (WHO) China Country Office. A novel beta-coronavirus, exhibiting 96% genomic identity with a previously detected SARS-like bat coronavirus (Zhou et al., 2020), was soon identified as the etiological agent and named SARS-CoV-2. On 30 January 2020, the WHO declared the epidemic a public health emergency of international concern, and soon after, on March 11th 2020, the SARS-CoV-2 pandemic was declared.

In Spain, SARS-CoV-2 was first detected in late January in the Canary Islands and soon after was detected in the Balearic Islands in an asymptomatic individual. Several other cases were reported in different Spanish regions since those first detections, however, sustained transmission events were not detected till March, moment in which the number of new notified cases exponentially grew and lockdown countermeasures were introduced by the Spanish Government. In the Balearic Islands, airports and ports were totally closed for regular passengers till May 2020, rendering the Islands a virtually closed environment for SARS-CoV-2 evolution. In summer 2020 airports and ports reopened and more than 10 M of tourists have arrived to some of the islands^1^. Of note, since October 2020, a negative test result for SARS-CoV-2 or a vaccine certificate has been required to entry.

A year and a half after the pandemic declaration, 230 million cases of COVID-19 have been reported worldwide, including 4.7 M deaths. More than 66 million of the cases have been reported in Europe, being Spain among the five European countries reporting more cases (4.9 M) [(European Centre for Disease Prevention and Control, 2021), data extracted on 23th September 2021].

Whole genome sequencing of SARS-CoV-2 has played a key role during this pandemic and is now established as an essential tool for making informed public health decisions, being key for identifying new emerging variants, as well as for monitoring their spread in communities and populations. In the Balearic Islands, more than 2,700 positive randomly selected respiratory samples have been fully sequenced since the onset of the pandemic in order to determine the viral genetic diversity of circulating variants. In this work, we report the genomic epidemiology of SARS-CoV-2 in a unique setting in which the course of the pandemic has been influenced by a complex interplay between insularity, severe social restrictions, and tourism travels.

## Materials and Methods

### Sampling

The Balearic Islands has four main islands: Majorca, the major island, with a total resident population of 912,171 people, Menorca with 95,641, Ibiza with 151,827 and Formentera with only 11,904 inhabitants. Since the first SARS-CoV-2 detection, 98,905 cases of COVID-19 have been reported in the Balearic Islands, being its distribution as follows: 73,034 cases reported in Majorca, 20,616 in Ibiza/Formentera and 5,252 in Menorca [(Servei de salut Illes Balears Conselleria Salut i consum, 2021), data extracted on 20th September 2021].

SARS-CoV-2 whole genome sequencing was set up in the Microbiology Department of the reference hospital of the Balearic Islands (Son Espases University Hospital). One day per week, all positive SARS-CoV-2 respiratory samples detected in the Microbiology Departments of all major public hospitals from the Balearic Islands were collected and stored frozen at −80°C for further studies.

**FIGURE 1:**
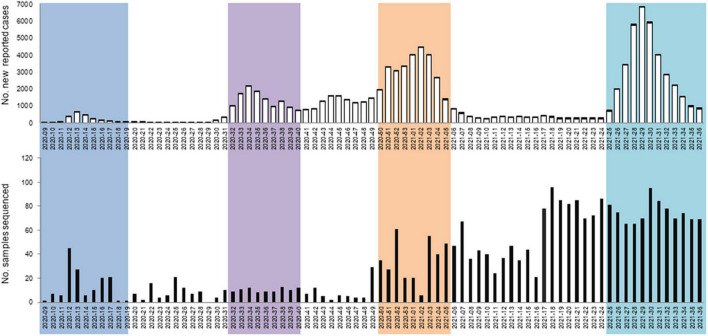
Number of new reported cases and samples selected for sequencing by epidemiological week. Epidemic waves in the Balearic Islands are indicated in colors: dark blue (1st epidemic wave), purple (2nd epidemic wave), orange (3rd epidemic wave), and light blue (4th epidemic wave).

Samples undergoing whole genome sequencing were randomly selected (*n* = 2,713), just considering the positivity rate in each island for its numerical distribution at the moment of sampling. Temporal distribution of positive respiratory samples selected for whole genome sequencing is shown in Figure 1. As shown, just one fifth of the samples (*n* = 553) sequenced were from 2020. In late 2020, lineages with higher numbers of spike mutations relative to previous circulating ones, emerged in different countries. The increased transmissibility and immune escape events associated with these emerging lineages definitely positioned Whole Genome Sequencing as an essential tool for monitoring the SARS-CoV-2 pandemic (European Center for Disease Prevention and Control, 2020; Ministerio de Sanidad, 2021). Thus, in order to accomplish the Spanish Ministry of Health and ECDC recommendations, sequencing capacities were gradually reinforced. SARS-CoV-2 whole genome sequencing was definitely included in the routine diagnostic procedures of the Microbiology Department of Son Espases University Hospital in May 2021, increasing sequencing capabilities to 95 samples per week.

### Genomic Library Preparation and Sequencing

RNA virus extraction from respiratory samples was performed with the MagMAX Viral/Pathogen II Nucleic Acid Isolation Kit (Thermo Fisher Scientific) which is specifically designed to recover RNA and DNA from viral particles contained in viral transport media.

Genomic library preparation was conducted following the SeqCOVID Consortium protocols (López et al., 2021). Briefly, RNA was first retro-transcribed into cDNA and SARS-CoV-2 complete genome amplification was conducted in two parallel multiplex PCR, accordingly to the openly available protocol developed by the ARTIC network (Quick, 2020) and using the V3 multiplex primers scheme (Github, 2019). Resulting amplicon pools were then combined and cleaned using AMPure beads (Beckman Coulter), and 50 ng were used to prepare the Illumina sequencing libraries (Illumina DNA Prep kit, Illumina Inc., San Diego, CA, United States) according to the manufacturer’s protocol and with 5 cycles for indexing PCR (NextEra DNA CD Indexes, Illumina). Finally, indexed genomic libraries were pooled in equimolar amounts and loaded on a MiSeq v3 cartridge (2 × 250 cycles).

### Lineage Assignment and Spike Mutation Surveillance

Two different bioinformatic approaches were used for whole genome sequence analysis: an open source pipeline based on IVAR (GitLab, 2020) and the DRAGEN COVID Lineage App available version (Illumina^®^). Both pipelines map quality- and primer- trimmed viral reads to the hCoV-19/Wuhan/WIV04/2019 reference sequence genome (MN908947.3/NC_045512.2) and result in the generation of consensus whole genome sequences.

Consensus sequences covering at least 75% of the reference sequence and with a median coverage greater than 100 reads were considered for lineage assignment. For this purpose, the Phylogenetic Assignment of Named Global Outbreak Lineages (PANGOLIN) tool was used (Github, 2020), employing the latest version and the most updated lineage database available at the time of assignment. Detection of additional spike amino acid substitutions was conducted by using the Basic Local Alignment Search Tool (BLAST) and the spike protein of the hCoV-19/Wuhan/WIV04/2019 strain as reference.

### Data Availability

Consensus SARS-CoV-2 genomic sequences were uploaded in the GISAID database, accession numbers of accepted sequences can be found in the Supplementary Material excel file.

## Results

### SARS-CoV-2 Genetic Diversity in the Balearic Islands

Whole genome sequencing was performed in 2,713 positive respiratory samples representing the 2.7% of the COVID-19 cases reported in the Balearic Islands; 2,050 samples were from Majorca (2.8%), 501 from Ibiza/Formentera (2.4%) and 162 from Menorca (3.1%).

Up to 2,297 of the generated consensus sequences (85%) were suitable for Pangolin lineage assignment. Whole genomes sequences were assigned to 46 different PANGO lineages (derivatives of lineages B.1.177 (B.1.177.X), B.1.526 (B.1.526.2), and B.1.617.2 (AY.X) were included within their parental lineage), and according to the WHO variants current definition, 75% of genomes were classified within a VOC, VUI, or VUM variant [World Health Organization [WHO], 2021]. Of note, 29 of the 46 PANGO lineages detected (63%) were assigned to less than 5 respiratory samples.

**FIGURE 2:**
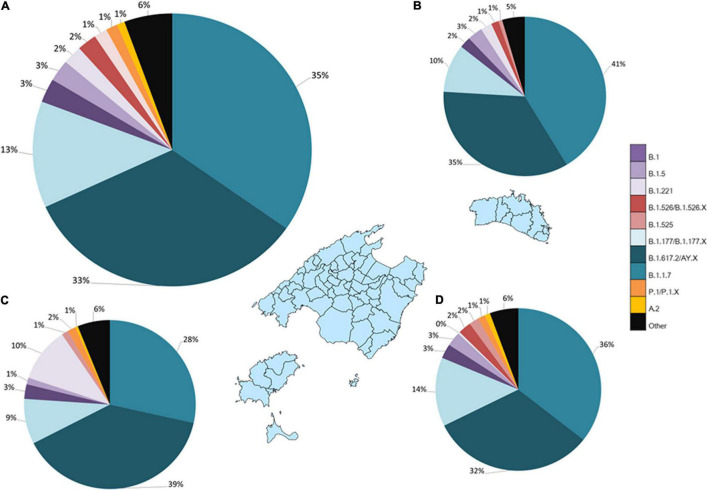
Geographical distribution of the 10 most frequently detected PANGO lineages. Most frequent lineages detected in **(A)** the Balearic Islands, **(B)** Menorca, **(C)** Ibiza/Formentera, and **(D)** Majorca are shown.

Globally, lineages B.1.1.7 (WHO Alfa variant) and B.1.617.2/AY.X (WHO Delta variant) have been identified as the 2 major lineages circulating in the Balearic Islands during the pandemic (35 and 33%, respectively), distantly followed by lineages B.1.177/B.1.177.X (13%). Top ten more prevalent lineages include lineages B.1 (2.8%), B.1.5 (2.6%), B.1.526/B.1.526.X lineages (WHO Iota variant; 2.3%), B.1.221 (2.1%), B.1.525 (WHO Eta variant; 1.5%), P.1/P.1.X (WHO Gamma variant; 1.3%) and A.2 lineage (0.9%) (Figure 2). VOC/VUM lineage B.1.351 (*n* = 9, WHO Beta variant) and VOI/VUM B.1.427/B.1.429 (*n* = 9, WHO Epsilon variant), B.1.619 (*n* = 1), B.1.620 (*n* = 1), B.1.1.238 (*n* = 2) and R.1 (*n* = 1) lineages have been rarely detected in the Balearic Islands (Supplementary Material).

Genetic diversity and distribution of circulating lineages were slightly different among islands. Highest genetic diversity was documented in the island of Majorca, where 42 different circulating lineages were detected, compared to Ibiza/Formentera (*n* = 18) and Menorca (*n* = 13). Of note, 24 of the 42 PANGO lineages were only detected in Majorca, including the B.1.621 lineage (WHO Mu variant). Distribution of circulating lineages was very similar in Majorca and Menorca but differ from Ibiza/Formentera, where the designated Eta variant (B.1.526/B.1.56.X) has not circulated and lineage B.1.221 was determined to be the third most prevalent lineage (10 vs. 9% lineage B.1.177) (Figure 2).

### Temporal Distribution of SARS-CoV-2 Major Lineages in the Balearic Islands

Since the beginning of the pandemic, four epidemic waves have been registered in the Balearic Islands: first was registered from late February 2020 to epidemiological week 2020-19, second from week 32 to 40-2020, third from week 50-2020 to 05-2021, and fourth from week 25 to 36-2021 (Servei de salut Illes Balears Conselleria Salut i consum, 2021).

**FIGURE 3:**
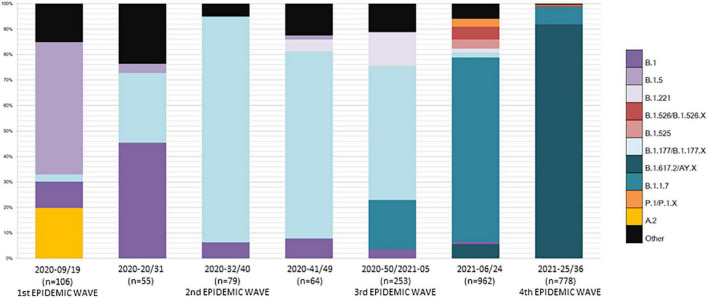
Temporal evolution of SARS-CoV-2 lineages. Lineage distribution in each of the epidemic waves (and intermediate periods) registered in the Balearic Islands.

Lineage A viruses (A.2 and A.5) were detected in all the Balearic Islands but only during the first epidemic wave, accounting for almost 25% of the sequenced samples in this period (Supplementary Material). As shown in Figure 3, the first epidemic wave was dominated by B.1 and B.1.5 lineages, although up to 12 different circulating lineages were detected (Supplementary Material).

Lineage B.1.177 dominated the second and third epidemic waves in the Balearic Islands; however, it was first detected in samples obtained in epidemiological week 17-2020. Of note, although the third epidemic wave was dominated by lineage B.1.177 (52.6% of the sequenced samples), up to 19.4 and 13% of the sequenced samples were assigned to the B.1.1.7 (WHO Alfa variant) and B.1.221 PANGO lineages.

After the third epidemic wave, sustained levels of transmission were documented in the Balearic Islands (Servei de salut Illes Balears Conselleria Salut i consum, 2021). During first half of 2021, the Alpha variant (PANGO lineage B.1.1.7) become the most prevalent circulating lineage (72.2%). Other lineages circulating during this period include the WHO Beta (P.1/P.1.X), Iota (B.1.525) and Eta (B.1.526/B.1526.X) variants, with frequencies of 3.1, 3.5, and 5%, respectively (Figure 3).

Finally, in summer 2021, designated PANGO lineage B.1.617.2 (WHO Delta variant) was first detected in Majorca. The Delta variant soon becomes dominant and most COVID-19 cases registered in the Balearic Islands during the fourth epidemic wave were ascribed to this lineage (Figure 3).

### Spike Mutations Surveillance

Most mutations encountered in the SARS-CoV-2 genome are expected to be either deleterious and swiftly purged or relatively neutral; however, a small proportion is supposed to affect the virus biology and may alter infectivity, disease severity or interactions with host immunity (Harvey et al., 2021). Since the emergence of lineages B.1.1.7, B.1.351, and P.1 harboring convergent and some preoccupant spike mutations, the presence of additional spike mutations different from those defining lineages has been routinely investigated in sequenced samples.

During first half of 2021, substitutions at position E484 in the spike protein, naturally occurring in B.1.351 and P.1 lineages, have been detected in 3 genome sequences ascribed to lineages B.1.1.7 (S:E484K, *n* = 2) and B.1.617.2 (S:E484G, *n* = 1). S:P681R-defining mutation of PANGO lineages B.1.617.2 and AY.X, have been detected in 2 genomes belonging to lineages B.1 and B.1.1.7. Moreover, up to 31 genomes ascribed to lineage B.1.1.7 harbored the spike substitution S:R287K, a rare mutation with unknown biological effect.

Of note, since July 2021, mutation S:Q613H present in lineage A.23.1 (Bugembe et al., 2021) has been detected in a relative high number of genomes belonging to the P.1 (*n* = 1) and B.1.617.2/AY.X (*n* = 24) lineages, mutation which could provide some biological advantages (Bugembe et al., 2021; Harvey et al., 2021). Additionally, in summer 2021, three consensus genome sequences were assigned to lineage B.1 (EPI_ISL_3087129, EPI_ISL_2626134, and EPI_ISL_2626162) and showed some interesting additional spike mutations, including S:L452R and S:E484Q; these three genomes have been reassigned to recently described lineage B.1.630.

## Discussion

Phylogenetic analyses have demonstrated that in Spain the epidemic started via multiple independent introductions of the virus, however, compared to other European countries, a large proportion of the earliest genomes were lineage A viruses (Díez-Fuertes et al., 2021; López et al., 2021). Lineage A viruses share two nucleotides with the closest known bat viruses (RaTG13 and RmYB02) and are related with the earliest SARS-CoV-2 detected in Wuhan, China (Rambaut et al., 2020a). In the Balearic Islands, these lineages were also detected but with a lower frequency than in the rest of Spain (Alm et al., 2020). Since early 2020, lineage A viruses have not been detected which probably reflects an evolutionary advantage of the D614G spike mutation present in all lineage B viruses (Hou et al., 2020; Yurkovetskiy et al., 2020; Volz et al., 2021).

As in the rest of the World, the SARS-CoV-2 epidemic in the Balearic Islands has been clearly dominated by lineage B viruses (Figures 1, 3). Lineages B.1 and B.1.5 were the most prevalent lineages detected during the first epidemic wave but, after this period, an increased frequency of B.1.177 was documented. This lineage, initially named 20E-EU1 variant and characterized by the spike substitution A222V, was identified in Spain in early summer 2020 and rapidly became the dominant lineage in several European countries (Hodcroft et al., 2021). B.1.177 was the most frequently detected lineage circulating during the second and the third epidemic waves. Of note, Hodcroft et al. (2021) did not find evidence of an increased transmissibility of viruses ascribed to this lineage and attribute the success of this lineage in the European countries to social behavior and summertime travels. Indeed, apart from Morocco and Tunisia, this lineage has been rarely detected outside Europe, and even the highest global daily prevalences (20%) (Latif et al., 2021), registered by the end of October 2020, were much lower than that reached in Spain (80%) and several other European countries (e.g., United Kingdom, Ireland, and Italy >60%; Denmark, Netherlands, and Norway >40%) (Hodcroft et al., 2021).

In comparison with the rest of the country and other European countries, in the Balearic Islands the third epidemic wave started earlier and was dominated by the most prevalent lineage at that moment, lineage B.1.177. However, almost one fifth of the samples sequenced in this period belong to the PANGO lineage B.1.1.7 (WHO Alpha variant). Lineage B.1.1.7 was first detected in early December, and soon after, it became the dominant circulating lineage, reaching similar prevalences to those registered in United Kingdom and other European countries (O’Toole et al., 2021b). This lineage is defined by 14 amino acid changes and three deletions, including six amino acid substitutions and two deletions in the spike protein: S:ΔH69–V70, S:ΔY144, S:N501Y, S:A570D, S:P681H, S:T716I, S:S982A, and S:D1118H (Rambaut et al., 2020b) and it has been related with some evolutionary advantages such as an increased transmissibility (Leung et al., 2021). From January to May 2021, severe social restrictions were applied in the Balearic Islands, restrictions that probably explain the sustained levels of virus transmission and COVID-19 cases registered during that period despite of the high prevalence of lineage B.1.1.7.

In late 2020 and early 2021, PANGO lineages B.1.351 and P.1 also received much attention mainly because of the presence of convergent mutations in the spike protein that could affect the biological characteristics of the virus (Harvey et al., 2021). These two lineages have been hardly detected by whole genome sequencing in the Balearic Islands. Moreover, since July 2021, PCR assays for detection of these variants have also been conducted in all new positive samples for SARS-CoV-2, being these lineages also rarely detected by this technique (data not shown). Altogether, these results discard sustained local transmission of these lineages in the Islands.

In mid-April, the United Kingdom government reported an increased in COVID-19 case numbers, hospitalizations and deaths despite ongoing vaccination programs and attributed the increased to a rise of PANGO lineage B.1.617.2 (WHO Delta variant) virus circulation (Callaway, 2021; Mahase, 2021). This lineage, first detected in India, also presented an unusual number of spike substitutions which have been linked to evolutionary advantages such as increased transmissibility and moderately resistance to vaccines, particularly in people who have received just a single dose (Lopez Bernal et al., 2021). In the Balearic Islands, lineage B.1.617.2 was first detected in early summer, and as it has occurred in the rest of Europe, North America and Asia (O’Toole et al., 2021a), rapidly displaced all other circulating variants including the Alpha variant. Since its introduction, the number of cases raised exponentially leading to the forth epidemic wave registered in the Islands. Although an increased transmissibility has been widely reported for this lineage; the number of registered cases may be also related with less social limitations and travels during summer 2021.

Tracking of potentially relevant spike protein mutations showed that additional mutations of those defining the lineage are not common among VOC/VUI/VUM lineages. Nevertheless, in late weeks, the additional spike substitution S:Q613H has been detected in an increasing number of genomes belonging to the Delta variant. This mutation is speculated to be important as it occurs at a position neighboring the fitness-enhancing mutation S:D614G (Bugembe et al., 2021).

Altogether, this work gives a snapshot of SARS-CoV-2 lineages circulating in the Balearic Islands. Results suggest that virus evolution, mainly driven by the acquisition and selection of spike substitutions conferring biological advantages, social restrictions and size population play a major role in the epidemic dynamics.

## Data Availability Statement

The datasets presented in this study can be found in online repositories. The names of the repository/repositories and accession number(s) can be found in the article/Supplementary Material.

## Author Contributions

CL-C conceived the study, performed the laboratory experiments and bioinformatics analysis, analyzed the results, and wrote the manuscript. PF-R, EB-T, MP, and JL performed the laboratory experiments. SJ-S and GC performed the bioinformatics analysis. AL, AH, EiR, ASe, EvR, LC, MF-B, CG, JS, and EA contributed materials. MS, ASa, and AN analyzed the results. FG-C and IC contributed laboratory protocols and analysis tools. AO conceived the study, analyzed the results, and wrote the manuscript. All authors critically reviewed the manuscript.

## Conflict of Interest

The authors declare that the research was conducted in the absence of any commercial or financial relationships that could be construed as a potential conflict of interest.

## Publisher’s Note

All claims expressed in this article are solely those of the authors and do not necessarily represent those of their affiliated organizations, or those of the publisher, the editors and the reviewers. Any product that may be evaluated in this article, or claim that may be made by its manufacturer, is not guaranteed or endorsed by the publisher.

## Footnotes

1
https://www.caib.es/
